# Impact of surface-area-to-volume ratio, internal viscosity and membrane viscoelasticity on red blood cell deformability measured in isotonic condition

**DOI:** 10.1038/s41598-019-43200-y

**Published:** 2019-05-01

**Authors:** Céline Renoux, Magalie Faivre, Amel Bessaa, Lydie Da Costa, Philippe Joly, Alexandra Gauthier, Philippe Connes

**Affiliations:** 1 Laboratoire Interuniversitaire de Biologie de la Motricité (LIBM) EA7424, Equipe “Biologie vasculaire et du globule rouge”, UCBL1 Lyon, France; 2 Laboratoire d’Excellence (Labex) GR-Ex, Paris, France; 3 Biochimie des pathologies érythrocytaires, Centre de Biologie et de Pathologie Est, HCL, Bron, France; 4 Université de Lyon; Institut des Nanotechnologies de Lyon INL-UMR5270 CNRS, Université Lyon 1, Villeurbanne, F-69622 France; 5 AP-HP, Service d’Hématologie Biologique, Hôpital R. Debré, Paris, F-75019 France; 6 Université Paris 7 Denis Diderot, Sorbonne Paris Cité, Paris, F-75010 France; 7 INSERM U1134, Paris, F-75015 France; 8 Institut d’hématologie et d’oncologie pédiatrique (IHOP), Hospices Civils de Lyon, Lyon, France; 9 Institut Universitaire de France, Paris, France

**Keywords:** Biophysics, Haematological diseases

## Abstract

Osmotic gradient ektacytometry is the gold standard to assess red blood cell (RBC) deformability. It has been proposed that, when measured in isotonic condition, RBC deformability at low shear stress would depend on membrane elasticity while it would be influenced by internal viscosity when measured at high shear stress, but this hypothesis needs to be further addressed. Healthy RBCs were rigidified by treatment with lysolecithine (LPC), diamide or nystatine associated with hyperosmolar solutions (OSMO), which reduces membrane surface area, decreases membrane elasticity or promotes cell dehydration, respectively. Diamide treatment resulted in a decrease in isotonic RBC deformability at all shear stresses tested (i.e. from 0.3 to 30 Pa). LPC and OSMO treatments caused a decrease in isotonic RBC deformability above 3 Pa only. Isotonic RBC deformability from patients with hereditary spherocytosis or sickle cell disease was mainly decreased above 1.69 Pa. Our findings indicate that decreased isotonic RBC deformability at shear stresses above 3 Pa would be related to a reduction in the surface-area-to-volume ratio and/or to a loss of membrane elasticity and/or to an increase in internal viscosity while a decrease of RBC deformability below 3 Pa would reflect a loss of membrane elasticity.

## Introduction

Red blood cells (RBCs) are discoid shape entities with a diameter of 8 µm and a thickness of 2 µm, which play a key role in gas transport in the human body. Indeed, every RBC needs to pass several times a day through narrow capillaries, without rupture, to release oxygen to the tissues. To accomplish this, RBCs need to dynamically adapt their shapes to the hemodynamic and vascular geometry conditions^1,2^. The deformability of a RBC is mainly influenced by three factors: (1) the surface-area-to-volume ratio (S/V; i.e., cell sphericity), (2) the internal viscosity, which is mainly dependent on the mean cell hemoglobin concentration (MCHC) and (3) the rheological properties of the membrane^3^. Although several methods have been proposed to characterize RBC deformability^4^, one of the most popular is the one initially developed by Bessis and Mohandas^5,6^ so called ektacytometry and in which a small amount of RBCs are suspended in a high viscous media and sheared into a Couette system. In that case, RBCs adopt an elliptic shape and elongate in the direction of the flow: the greater the elongation (reflected by the elongation or deformability index; i.e., EI or DI), the higher the deformability of RBCs.

Osmotic gradient ektacytometry is acknowledged as the reference technique to diagnose the most frequent RBC membrane disorders, such as hereditary spherocytosis (HS), elliptocytosis and its severe form, pyropoikilocytosis, south asian ovalocytosis and hereditary stomatocytosis^3,7–9^. It consists of shearing the RBC suspension at a constant shear stress with tonicity of the mixture varying between 50 and 600 mOsmol/kg during the measurement. Different parameters may be derived from ektacytometry curve, which provides information about RBC osmotic fragility, cell surface area, cell volume, RBC hydration state and RBC membrane deformability^3,7–9^.

While osmotic gradient ektacytometry is often used by hematologist involved in the screening of RBC membrane disorders, it is less frequently used by researchers involved in the field of blood rheology^10^ who prefers assessing RBC deformability with ektacytometer used at various shear stresses but under constant tonicity of the medium (i.e., isotonicity). This situation may be partly explained by the fact that osmotic gradient ektacytometers have not been easily available in the markets from the nineties to recently^8^. Moreover, the interpretation of osmotic gradient ektacytometry is less easy than for isotonic ektacytometry where most of the studies simply concluded about the status of RBC deformability in a given disease/situation (decreased, similar or increased) compared to a control population/situation^10^. Isotonic ektacytometry is indeed frequently used for RBC deformability measurement in metabolic disorders^11–13^, cardiovascular diseases^14–17^, ageing^18,19^ or hemoglobin diseases^9,20,21^. However, the exact meaning of the analysis performed in isotonic condition is unclear. The previous study conducted by Mohandas and colleagues^1^ suggested that changes in S/V would not affect isotonic RBC deformability while increased internal viscosity or a loss of membrane viscoelasticity would result in a decrease of isotonic RBC deformability. However, there is no clear information about the shear stress values at which these factors could affect isotonic RBC deformability or not. Later on, it has been suggested that decreased RBC deformability at low shear stresses would reflect impaired membrane viscoelasticity while RBC deformability at high shear stresses would be mainly affected by S/V ratio and/or the internal viscosity^10^. However, experiments are lacking to prove this assumption. The aim of the present study was to test these hypotheses. Indeed, we tested the effects of several molecules known to modulate surface area, cell volume, internal viscosity or membrane elasticity on healthy RBCs and we compared the RBC deformability measurements obtained with osmotic gradient ektacytometry versus isotonic ektacytometry. In addition, we used RBC from patients with HS or sickle cell disease (SCD) and compared the RBC deformability findings obtained with osmotic gradient ektacytometry versus isotonic ektacytometry to get further information on the meaning of isotonic ektacytometry results.

## Materials and Methods

### Samples

Blood samples from 5 healthy individuals, 3 patients with HS and 4 SCD patients were collected in EDTA tubes. Informed consent was obtained for every individual. The protocol was approved by the “Hospices Civils de Lyon – CPP Est” Ethics Committee (L14-127) and was performed in accordance with the guidelines set by the declaration of Helsinki. Healthy RBCs were treated with one of the following molecules to modulate their deformability: lysolecithin (LPC), nystatin associated with hyperosmolar solutions (OSMO) or diamide. RBCs were washed 2 times with phosphate buffered saline (PBS) 1X before and after each treatment, and then re-suspended at 40% hematocrit (Ht) in PBS 1X.

### Cell surface and S/V reduction (LPC treatment)

As previously described^1^, washed RBCs were incubated for 5 min at room temperature with LPC (Sigma-Aldrich, Saint-Louis, Missouri, United-States) at final concentrations of 0, 0.5 and 1.0 µmol/mL cells, to reduce the S/V ratio. LPC changed discocyte RBCs to echinocytes III, induced membrane vesiculation and loss of membrane surface area^1^.

### Membrane deformability reduction (diamide treatment)

Washed RBCs were incubated with diamide (Sigma-Aldrich, Saint-Louis, Missouri, United-States) at final concentrations of 0, 0.25 and 0.5 mmol/L for 1 h at 37 °C^1^. Diamide induced the formation of disulfide bonds between spectrin proteins and increased the shear modulus of RBCs^22^.

### Increase in internal viscosity (nystatin treatment with changes in osmolality; OSMO)

As previously described^1^, a suspending medium was prepared such as Na^+^ and K^+^ concentrations were those found in RBCs (Na = 12 mEq/L; K = 155 mEq/L). Then, the medium was divided in several parts and sucrose was added to obtain a final osmolality of 300, 400 or 500 mOsm/kg. Washed RBCs were re-suspended at 5% hematocrit in the different solutions. Nystatin (5 mg/mL methanol) was added to RBC suspensions to reach a final concentration of 30 µg/mL. Nystatin is an antibiotic, which allows the equilibration of Na^+^ and K^+^ across the cell membrane. After 20 min of incubation at 0 °C, nystatin was washed out of the membrane at 37 °C by solutions identical to the suspending medium but without nystatin to restore normal permeability characteristics to the cells^1^.

### RBC deformability

RBC deformability, reported as the elongation index (EI), was determined at 37 °C by using (1) osmotic gradient ektacytometry and (2) isotonic ektacytometry (Lorrca® MaxSis; RR Mechatronics, Hoorn, The Netherlands). A small amount (20 μl) of each RBC suspension was resuspended in high viscous medium (polyvinylpyrrolidone; PVP; viscosity = 30 cP) at 2% hematocrit and sheared into a Couette system made of glass. The diffraction pattern was recorded by a camera and EI was calculated based on the width and height of the theoretical ellipse fitted on the diffraction pattern^23^. Osmotic gradient ektacytometry measured EI of the RBC suspension under a defined shear stress (30 Pa) and at increasing osmolality (90 to 600 mOsm/kg) to determine several parameters: O_min_ (i.e., the osmolality at which RBC deformability value reaches a minimum in the hypotonic region of the curve), EI_max_ (i.e., the highest RBC deformability) and O_hyper_ (also called O’), which corresponds to the osmolality at half of the EI_max_ in the hypertonic region of the curve^3^. O_min_ reflects the osmotic fragility and the S/V ratio, EI_max_ depends on the membrane deformability and RBC surface area, and O_hyper_ reflects MCHC and cell volume^3,9^. In addition, RBC deformability measured at low osmolality is affected by the membrane flexibility^3,9^. Isotonic ektacytometry was used to measure EI at fixed isotonicity and at 9 increasing shear stresses (from 0.3 to 30 Pa). Mean cell volume (MCV) and MCHC were determined using an automated hematology analyzer (XN-10, Sysmex corp, Kobe, Japan).

### Statistical analysis

Results are presented as median [25th–75th] percentiles. A non-parametric Friedman ANOVA test for repeated measurement was used to compare the different parameters after cells treatment. The significance level was defined as p < 0.05. Analyses were conducted using SPSS software (v.22; IBM SPSS Statistics, Chicago, IL).

## Results

### Molecules treatment

**Figure 1:**
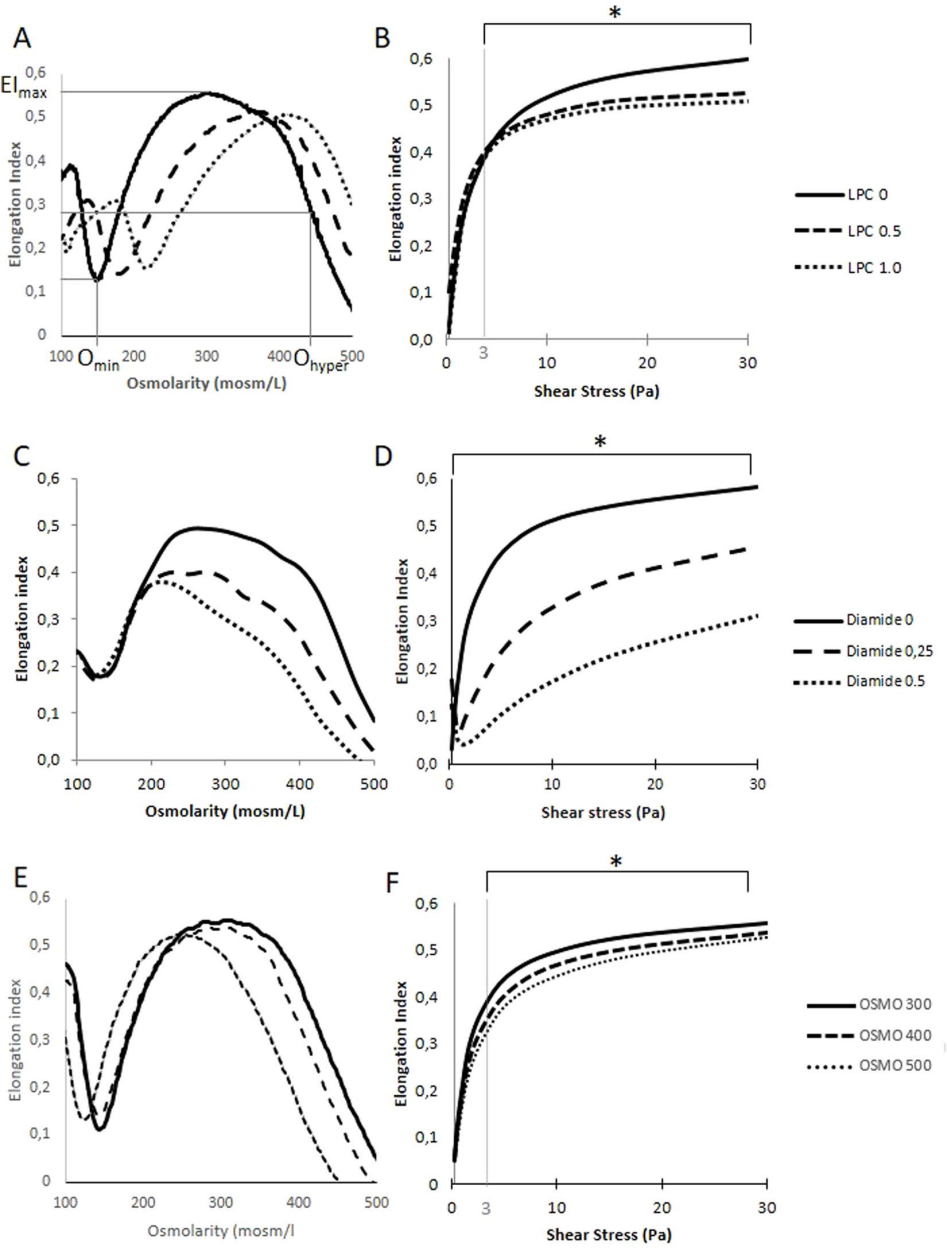
Osmotic gradient ektacytometry (**A**,**C**,**E**) and isotonic ektacytometry (**B**,**D**,**E**) profiles after LPC, diamide or nystatin + hyperosmolar (OSMO) treatments. In osmotic gradient conditions, LPC treatments caused a significant rise in O_min_ and O_hyper_ and a decrease in EI_max_ (**A**), and a decrease in EI above 3 Pa in isotonic conditions (**B**). Diamide treatments decreased EI_max_ and O_hyper_ in osmotic gradient conditions (**C**) and decreased EI at all shear stresses in isotonic conditions (**D**). In osmotic gradient conditions, OSMO treatments caused a decrease in O_min_ and O_hyper_. (**E**) and resulted in a decrease in EI above 3 Pa in isotonic conditions (**F**). Key parameters of osmotic gradient ektacytometry are also indicated on the (**A**): O_min_ is the osmolality at which RBC deformability value reaches a minimum in the hypotonic region of the curve, EI_max_ is the highest RBC deformability and O_hyper_ corresponds to the osmolality at half of the EI_max_ on the hypertonic region of the curve. O_min_ reflects the osmotic fragility and the S/V ratio, EI_max_ depends on the membrane deformability and RBC surface area, and O_hyper_ reflects MCHC and cell volume. Statistical significance: *p < 0.05.

Results of osmotic RBC deformability and hematological parameters after different RBC treatments are presented in the Table 1. LPC treatment caused a significant rise in O_min_ and a decrease in EI_max_ (Fig. 1A). We also observed a tendency for MCV to increase (p < 0.1) and MCHC to decrease (p < 0.1) with LPC, resulting in a significant increase in O_hyper_. In isotonic conditions, LPC caused a decrease in EI above 3 Pa but not below (Fig. 1B). Diamide treatment decreased EI_max_ and O_hyper_ (Fig. 1C). The increase in diamide concentration caused asymmetry in the hump of the osmotic gradient ektacytometry curve with a greater reduction in EI on the hypertonic part of the curve than on the hypotonic side. EI measured in isotonic conditions decreased at all shear stresses (Fig. 1D). OSMO caused a decrease in MCV, O_min_ and as expected O_hyper_, and an increase in MCHC. No change was observed for EImax (Fig. 1E). RBC treated with OSMO and studied in isotonic conditions showed a decrease in EI above 3 Pa but not below (Fig. 1F).

**Table 1:** Osmotic RBC deformability (Omin, EImax and Ohyper) and hematological parameters (mean cell volume, MCV; mean corpuscular hemoglobin concentration, MCHC) after LPC, diamide or nystatin + hyperosmolar (OSMO) conditions.

	LPC 0	LPC 0.5	LPC 1	*p*
**Omin (mosm/L)**	**148 [142; 149]**	**193 [186; 212]**	**220 [203; 229]**	*
**EImax**	**0.56 [0.54; 0.56]**	**0.51 [0.44; 0.52]**	**0.44 [0.38; 0.51]**	*
**Ohyper (mosm/L)**	**443 [437; 451]**	**485 [483; 485]**	**513 [511; 514]**	*
MCV (fL)	87.8 [87.1; 88.5]	105.2 [104.2; 105.2]	103.4 [94.7; 107.4]	*NS*
MCHC (g/L)	333 [315; 333]	273 [265; 280]	276 [261; 300]	*NS*
	**DIAMIDE 0**	**DIAMIDE 0.25**	**DIAMIDE 0.5**	***p***
Omin (mosm/L)	137 [123; 146]	132 [121; 140]	130 [122; 135]	*NS*
**EImax**	**0.51 [0.50; 0.51]**	**0.42 [0.39; 0.45]**	**0.39 [0.37; 0.41]**	*
**Ohyper (mosm/L)**	**450 [445; 460]**	**416 [407; 436]**	**388 [376; 400]**	*
MCV (fL)	92.5 [90.1; 97.3]	92.4 [87.6; 97.2]	88.2 [86.7; 91.6]	*NS*
MCHC (g/L)	310 [301; 314]	323 [320; 326]	324 [309; 329]	*NS*
	**OSMO 300**	**OSMO 400**	**OSMO 500**	***p***
**Omin (mosm/L)**	**147 [118; 163]**	**139 [111; 142]**	**120 [104; 124]**	*
EImax	0.52 [0.44; 0.54]	0.51 [0.45; 0.53]	0.52 [0.49; 0.53]	*NS*
**Ohyper (mosm/L)**	**437 [377; 482]**	**415 [340; 418]**	**326 [281; 369]**	**
**MCV (fL)**	**98.5 [96.3; 105.5]**	**85.6 [83.3; 92.1]**	**79.7 [78.4; 81.3]**	**
**MCHC (g/L)**	**300 [292; 308]**	**346 [337; 350]**	**362 [356; 371]**	**

Statistical significance: NS, non significant; *p < 0.05; **p < 0.01.

### Patient samples

**Figure 2:**
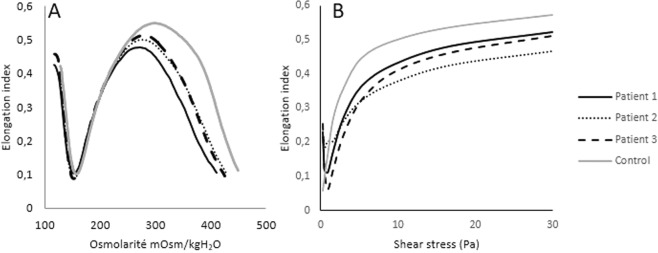
Osmotic gradient ektacytometry (**A**) and isotonic ektacytometry (**B**) profiles in patients affected by hereditary spherocytosis. In osmotic conditions, O_min_ was increased and O_hyper_ and EI_max_ decreased (**A**). In isotonic conditions, EI was decreased above 1.69 Pa but not below (**B**).

Osmotic gradient ektacytometry showed that HS patients had decreased O_hyper_ and EI_max_ compared to healthy subjects (Fig. 2A). O_min_ was in the normal range as it may sometimes occur in HS patients^7^. In isotonic conditions, EI is mainly decreased above 1.69 Pa but not below (Fig. 2B). At very low shear stresses (i.e., 0.3 and 0.53 Pa), EI of HS patients was higher than in normal individuals. In addition, EI measured in HS patients decreased between 0.03 and 0.95 Pa and then increased with the rise of shear stresses while normal RBCs showed a gradual increase with shear stresses from 0.3 to 30 Pa.

**Figure 3:**
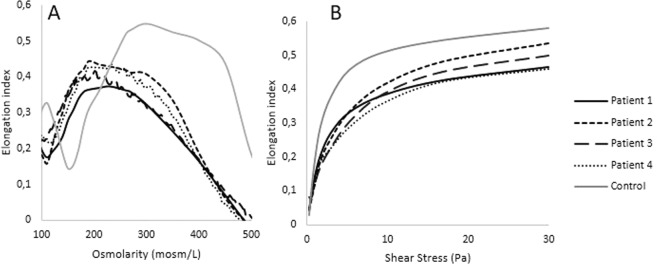
Osmotic gradient ektacytometry (**A**) and isotonic ektacytometry (**B**) profiles in patients affected by sickle cell disease. O_min_, EI_max_ and O_hyper_ were decreased in SCD patients (**A**). In isotonic conditions, EI was decreased above 0.95–1.69 Pa (**B**).

O_min_, EI_max_ and O_hyper_ were significantly decreased in the four SCD patients compared to healthy RBCs (Fig. 3A). When measured in isotonic condition, EI was decreased in the four SCD patients at shear stresses above 0.95–1.69 Pa (Fig. 3B).

## Discussion

Osmotic gradient ektacytometry allows the identification of cellular abnormalities responsible for a reduction of RBC deformability such as decreased membrane visco-elasticity, and/or changes in surface to volume ratio and/or cell dehydration. Indeed, this technique is frequently used by hematologists to diagnose several RBC membrane disorders. Our study demonstrates that a simple isotonic ektacytometry may also provide important information regarding RBC biophysical properties.

As previously shown, LPC increased O_min_ and decreased EI_max_, which is consistent with a reduction in the surface area of RBC^1,3^. However, while Mohandas *et al*.^1^ and Clark *et al*.^3^ reported no change in cell volume with LPC, we observed a rise in O_hyper_, which is consistent with the tendency for MCV to increase and MCHC to decrease. Increased RBC volume with LPC has also been reported in the study of Safeukui *et al*.^24^. The consequences of the decrease in S/V ratio (i.e., increased cell sphericity) were also observed in isotonic conditions where deformability measured at 3 Pa and higher shear stresses was lower in RBCs treated with LPC than in control RBCs. Nevertheless, the change in S/V ratio with LPC had no consequence on RBC deformability measured at a shear stress below 3 Pa. The use of nystatin and hyperosmolar conditions to increase the internal viscosity also resulted in a reduction of RBC deformability measured in isotonic condition at 3 Pa and higher shear stresses. Nevertheless, it should be noted that the magnitude of changes for isotonic RBC deformability measured above 3 Pa was slightly higher with LPC than with OSMO. The degree of isotonic RBC deformability impairment probably depends on the number of dense dehydrated RBCs present in the blood suspension, as it is the case in sickle cell disease^25^ and hereditary spherocytosis^8^. Nystatin used with higher osmolalities could result in a larger decrease in RBC deformability. As previously reported, RBCs treated with diamide were characterized by decreased EI_max_ and O_hyper_ and enhanced curve asymmetry with a strong reduction of deformability in the hypertonic part^1,3^. Diamide is known to increase the shear modulus of RBCs because of the cross-linking between spectrins^22^. As a consequence, isotonic RBC deformability was reduced at all shear stresses.

The curves obtained with the osmotic gradient ektacytometry for HS and SCD patients were typical of these two diseases^3,8^. Isotonic RBC deformability for HS patients showed a decrease at shear stresses above 1.69 Pa. The isotonic curves of these patients look like a mix of the isotonic curves observed with LPC- and OSMO-treated cells, where cell sphericity and dehydration are increased, respectively. This pattern is in agreement with the slightly increased O_min_ and the decreased EI_max_ and O_hyper_ observed in the HS studied patients, which demonstrate a decrease in the S/V ratio, a loss of surface area and dehydration of the cells, respectively^3,8^. The comparisons of the osmotic gradient ektacytometry and isotonic curves for HS patients demonstrate that it is easier to characterize the RBC properties with the first method, which confirms that osmotic gradient ektacytometry gives more information on the factors affecting RBC deformability than isotonic ektacytometry. Nevertheless, our results demonstrate that measurement of isotonic RBC deformability may also be useful to suspect RBC membrane disorders. The higher isotonic RBC deformability found at very low shear stresses could be related to the presence of highly dehydrated RBCs, which could have difficulties to align into the flow direction in these hemodynamic conditions but further studies are needed to better understand the meaning of this phenomenon. In the case of SCD patients and as already reported^3^, we observed a reduction in osmotic fragility as well as a shift of O_hyper_ to lower osmolalities indicating the presence of dehydrated RBCs. The reduction in EI_max_ was highly variable from one patient to another, which is in agreement with the findings of Clark *et al*.^3^ who showed heterogeneous EI_max_ values in this disease because of high heterogeneity in cell water content rather than a reduction in surface area. The reduction in isotonic RBC deformability seems to be higher in SCD than in HS patients but also more heterogeneous, which may be explained by the presence of variable number of dense RBCs in the blood of SCD patients^25^. In addition, increased shear modulus caused by the interaction between sickle hemoglobin and the RBC membrane have been demonstrated to participate to the reduction in RBC deformability in SCD^26^.

In conclusion, our results indicate that decreased isotonic RBC deformability at shear stresses above 3 Pa would be related to a reduction in S/V ratio and/or to a loss of membrane flexibility and/or to an increase in internal viscosity while a decrease of RBC deformability below 3 Pa would reflect a loss of membrane flexibility only. Indeed, for researchers using only isotonic ektacytometry, interpreting the whole curve with RBC deformability measured at both low-moderate and high shear stresses may be useful to better understand the reasons of impaired RBC deformability in a given population or experimental situation. Nevertheless, although isotonic ektacytometry seems to be helpful to suspect the presence of RBC membrane or hemoglobin disorders, osmotic gradient ektacytometry and complementary laboratory/molecular tests are needed to make the diagnosis^8^.
